# Rutin-Loaded Nanostructured Lipid Carriers Attenuate Doxorubicin-Induced Nephrotoxicity and Modulate Epigenetic Regulation and MyD88/STAT3 Signaling

**DOI:** 10.3390/toxics14080713

**Published:** 2026-08-12

**Authors:** Amina A. Farag, Walaa Bayoumie El Gazzar, Mahmoud Mostafa, Lina A. Mohammed, Azza S. El-Demerdash, Nagah E. M. Ali, Ranih Z. Amer, Heba S. Youssef, Noha Osama El-Shaer, Ibrahim A. Mostafa, Haidy M. Fakher, Sahar Soliman

**Affiliations:** 1 Department of Forensic Medicine and Clinical Toxicology, Faculty of Medicine, Benha University, Benha 13518, Egypt; amina.farag@fmed.bu.edu.eg (A.A.F.); nageh.ali@fmed.bu.edu.eg (N.E.M.A.); ibrahim.attai@fmed.bu.edu.eg (I.A.M.); haydi.abdelsalam@fmed.bu.edu.eg (H.M.F.); 2 Department of Anatomy, Physiology and Biochemistry, Faculty of Medicine, The Hashemite University, Zarqa 13133, Jordan; walaa.algazar@fmed.bu.edu.eg; 3 Department of Medical Biochemistry & Molecular Biology, Faculty of Medicine, Benha University, Benha 13518, Egypt; lina.mohamed@fmed.bu.edu.eg; 4 Department of Pharmaceutics, Faculty of Pharmacy, Minia University, Minia 61519, Egypt; mahmoud_mohamed@mu.edu.eg; 5 Department of Pharmaceutics, Faculty of Pharmacy, Minia National University, New Minia 61768, Egypt; 6 Department of Biotechnology, Agricultural Research Center (ARC), Animal Health Research Institute (AHRI), Zagazig Branch, Zagazig 44516, Egypt; dr.azzasalah@yahoo.com; 7 Department of Oral Surgery and Diagnostic Sciences, Applied Science Private University (ASU), Amman 11937, Jordan; r_suliman@asu.edu.jo; 8 Department of Physiology, Faculty of Medicine, Benha University, Benha 13518, Egypt; heba.youssef@fmed.bu.edu.eg (H.S.Y.); noha.saad@fmed.bu.edu.eg (N.O.E.-S.); 9 Department of Physiology and Pharmacology, College of Osteopathic Medicine, Sam Houston State University, Conroe, TX 77304, USA

**Keywords:** doxorubicin, kidney injury, epigenetics, klotho methylation, MyD88/STAT3, nanostructured lipid carriers, rutin, oxidative stress

## Abstract

Doxorubicin (DOX)-induced nephrotoxicity remains a major limitation to its clinical use, yet the underlying epigenetic mechanisms are incompletely understood. This study investigated the role of epigenetic dysregulation and MyD88/STAT3 signaling in DOX-induced renal injury and evaluated the renoprotective efficacy of rutin-loaded nanostructured lipid carriers (RUT-NLCs) compared with free rutin (RUT). Thirty-six rats were allocated to six experimental groups, and renal function, oxidative stress, inflammation, DNA damage, epigenetic modifications, MyD88/STAT3 signaling, histopathology, and ultrastructural changes were assessed. DOX administration induced severe renal dysfunction, oxidative stress, DNA damage, tubular injury, global DNA hypermethylation, aberrant histone methylation, Klotho promoter hypermethylation, and activation of the MyD88/STAT3 inflammatory pathway. Treatment with RUT-NLCs significantly attenuated these alterations by restoring antioxidant defenses, normalizing epigenetic markers, reducing DNA damage, suppressing MyD88/STAT3 signaling, and improving renal histopathological and ultrastructural architecture. Overall, RUT-NLCs provided greater nephroprotection than free rutin, suggesting that modulation of epigenetic alterations and MyD88/STAT3 signaling represents a key mechanism underlying their therapeutic efficacy against DOX-induced nephrotoxicity.

## 1. Introduction

Acute kidney injury (AKI) is a serious condition associated with substantial morbidity and mortality, accounting for approximately 2 million deaths worldwide each year. Doxorubicin (DOX), a potent anthracycline chemotherapeutic agent, is widely used in the treatment of various malignancies, including breast cancer, lymphomas, and sarcomas [1]. DOX-induced nephropathy is recognized as one of the leading causes of chemotherapy-associated AKI. Notably, DOX is primarily metabolized in the liver and partially excreted by the kidneys; therefore, renal injury may further exacerbate its systemic toxicity by impairing drug elimination [2]. Accordingly, elucidating the molecular mechanisms underlying DOX-induced AKI is essential for identifying effective renoprotective strategies.

Accumulating evidence indicates that oxidative stress plays a central role in DOX-induced nephrotoxicity. DOX-iron complexes promote the generation of reactive oxygen species (ROS), including hydroxyl radicals, hydrogen peroxide, and superoxide anions, thereby contributing to renal injury [1]. Excessive ROS cause extensive damage to cellular macromolecules, including lipids, proteins, and nucleic acids, while disrupting the mitochondrial electron transport chain. These effects lead to mitochondrial dysfunction, DNA damage, and impaired cellular homeostasis [3]. Because of their high metabolic activity and abundant mitochondrial content, renal tubular cells are particularly susceptible to oxidative injury [1]. Persistent oxidative stress subsequently activates multiple downstream signaling pathways involved in inflammation and apoptosis [3], leading to increased production of pro-inflammatory mediators such as tumor necrosis factor-α (TNF-α), which further amplifies renal inflammation and tissue injury [4].

Growing evidence indicates that epigenetic mechanisms play a significant role in both AKI and the kidney’s repair processes [5]. Epigenetic regulation refers to heritable changes in gene expression that occur without alterations in the underlying DNA sequence [6]. Among these mechanisms, DNA methylation and histone modifications have emerged as key regulators of gene expression programs involved in renal inflammation, oxidative stress, and fibrosis during AKI [7].

Klotho is a kidney-derived protein with well-established renoprotective properties, including anti-inflammatory and antifibrotic effects [8]. Because the kidney is the primary source of Klotho production, its expression is markedly reduced in various kidney diseases [9]. Importantly, epigenetic dysregulation of the Klotho gene, particularly promoter hypermethylation, has been implicated in the loss of Klotho expression in renal disease [10]. Therefore, targeting reversible epigenetic alterations may represent a promising therapeutic strategy to mitigate nephrotoxicity and promote renal recovery.

Myeloid differentiation primary response 88 (MyD88) is an adaptor protein that links extracellular signals to intracellular signaling pathways and serves as a key mediator of innate immune responses. Following Toll-like receptor 4 (TLR4) activation, MyD88 is recruited to initiate downstream signaling cascades [11]. Activation of the TLR4/MyD88 axis has been associated with downstream JAK/STAT3 signaling and NF-κB activation [12], contributing to inflammatory and apoptotic responses in experimental models of AKI.

Given the complex pathogenesis of DOX-induced AKI, there is a pressing need for therapeutic strategies that target oxidative stress, inflammation, and epigenetic dysregulation. Rutin (RUT), a naturally occurring flavonoid glycoside, possesses potent antioxidant and anti-inflammatory properties; however, its therapeutic application is limited by poor aqueous solubility and low bioavailability [13,14]. Nanostructured lipid carriers (NLCs) have emerged as promising drug-delivery systems because of their high drug-loading capacity, ability to improve drug dispersion and protection, and controlled-release characteristics [15]. Encapsulation of RUT within NLCs may therefore improve its pharmacokinetic profile and therapeutic efficacy in AKI [16].

Although oxidative stress and inflammation are recognized contributors to DOX-induced nephrotoxicity, the relationship among epigenetic alterations, Klotho regulation, and key inflammatory signaling pathways remains incompletely understood. In particular, the association among Klotho methylation, histone modifications, and MyD88/STAT3 signaling during DOX-induced AKI has not been fully defined. Moreover, while RUT is known for its antioxidant and anti-inflammatory properties, its ability to modulate epigenetic alterations in DOX-induced renal injury remains unclear. Therefore, the present study investigated the renoprotective effects of RUT, particularly when delivered through NLCs, and examined its effects on epigenetic alterations and MyD88/STAT3 signaling in DOX-induced AKI.

## 2. Materials and Methods

### 2.1. Drug and Chemicals

Doxorubicin (DOX) was purchased from EIMC United Pharmaceuticals (Badr City, Cairo, Egypt). Rutin (RUT) was obtained from SDFCL S.D. Fine-Chem Ltd. (Mumbai, India). Stearic acid, oleic acid, Span 60, and all other chemicals and solvents, unless otherwise specified, were purchased from El-Nasr Pharmaceutical Company (Cairo, Egypt) and were of analytical grade.

### 2.2. Animals and Ethical Approvals

#### 2.2.1. Animals

Thirty-six adult male Wistar albino rats (6–8 weeks old, 160–180 g) were obtained from the Faculty of Veterinary Medicine, Benha University (Moshtohor, Egypt). Animals were randomly assigned to six experimental groups (*n* = 6 per group). Rats were housed under standard laboratory conditions (25 ± 2 °C, 45 ± 5% relative humidity, and a 12 h light/12 h dark cycle) with free access to standard chow and water. All animals were acclimatized for one week prior to the start of the experiment.

All experimental procedures were conducted in accordance with institutional and international guidelines for the care and use of laboratory animals and were approved by the Research Ethics Committee of Minia University, Egypt (Approval No. MPEC 250601).

#### 2.2.2. ARRIVE Statement

The study was conducted in accordance with the Guide for the Care and Use of Laboratory Animals (NIH Publication No. 8023, revised 1978) and complied with the ARRIVE guidelines.

### 2.3. Methods

#### 2.3.1. Preparation of RUT-Loaded Nanostructured Lipid Carriers (RUT-NLCs)

RUT-loaded nanostructured lipid carriers (RUT-NLCs) were prepared using the hot high-shear homogenization method with minor modifications [17]. Briefly, a mixture of stearic acid (solid lipid) and oleic acid (liquid lipid) at a 2:1 (*w*/*w*) ratio was heated to 75 °C until a clear molten phase was obtained. Span 60 (2%, *w*/*w*) and RUT were then added to the molten lipid phase under continuous magnetic stirring until completely dissolved.

The aqueous phase was heated separately to the same temperature and gradually added to the lipid phase under high-shear homogenization at 15,000 rpm for 5 min to form a coarse emulsion. The resulting emulsion was allowed to cool gradually to room temperature, leading to solidification of the lipid matrix and formation of RUT-NLCs.

Unencapsulated RUT was separated by centrifugation at 15,000 rpm for 60 min. The supernatant was collected, and the concentration of unencapsulated drug was determined by UV-Vis spectrophotometry at 272 nm. The entrapment efficiency (EE%) was calculated by quantifying the unentrapped RUT spectrophotometrically, using the following equation:

$$EE\%=\frac{Total\;RUT\;amount-Free\;RUT\;amount}{Total\;RUT\;amount}\times 100$$

The mean particle size, polydispersity index (PDI), and zeta potential of the prepared NLCs were characterized by dynamic light scattering and electrophoretic light scattering at 25 °C following appropriate dilution with distilled water using Zetasizer Nano (Malvern, UK).

The surface morphology of the prepared RUT-NLCs was evaluated using scanning electron microscopy (SEM) (JEOL JSM-6510LV, JEOL Ltd., Tokyo, Japan). Briefly, a diluted sample of the RUT-NLC dispersion was deposited onto a clean glass coverslip and allowed to dry at room temperature. The dried sample was mounted onto an aluminum stub using double-sided carbon adhesive tape and sputter-coated with a thin gold layer to enhance electrical conductivity and imaging quality. The morphology, surface characteristics, and shape of the nanoparticles were examined using SEM at an appropriate accelerating voltage. Representative SEM images were obtained to evaluate the morphological features and surface architecture of the optimized RUT-NLC formulation.

In vitro drug release was assessed using a dialysis bag diffusion approach. Briefly, formulations were placed in dialysis bags and immersed in phosphate-buffered saline (pH 7.4) supplemented with 10% ethanol, maintained at 37 ± 0.5 °C under continuous shaking (60 rpm). Aliquots were withdrawn at predefined time points over 24 h, filtered, and quantified by UV-Vis spectrophotometry at 272 nm to determine RUT release. Cumulative drug release (%) was plotted as a function of time. Experiments were conducted in triplicate, and data are presented as mean ± SD.

#### 2.3.2. Experimental Design

Rats were randomly assigned to six experimental groups (*n* = 6 per group):

Control group (Ctrl): Rats received intraperitoneal (i.p.) injections of saline daily for 14 days.

RUT group (RUT): Rats received RUT (10 mg/kg, i.p.) every other day for 10 days [18].

RUT-NLCs group (RUT-NLCs): Rats received RUT-NLCs (10 mg/kg, i.p.) every other day for 10 days.

DOX group (DOX): Rats received a single intraperitoneal injection of DOX (20 mg/kg) [19].

DOX + RUT group (DOX-RUT): Rats received RUT (10 mg/kg, i.p.) every other day for 10 days, followed by a single intraperitoneal injection of DOX (20 mg/kg).

DOX + RUT-NLC group (DOX-RUT-NLCs): Rats received RUT-NLCs (10 mg/kg, i.p.) every other day for 10 days, followed by a single intraperitoneal injection of DOX (20 mg/kg).

#### 2.3.3. Sample Collection and Tissue Preparation

Blood samples (2 mL) were collected from the retro-orbital venous plexus and allowed to clot at room temperature for 15–30 min before centrifugation at 3000× *g* for 10 min at 4 °C. Serum was separated and stored at −20 °C for subsequent assessment of renal function biomarkers. Following blood collection, animals were euthanized by decapitation under anesthesia.

Both kidneys were excised, rinsed with ice-cold physiological saline to remove residual blood, and divided into several portions. One portion was fixed in 10% neutral-buffered formalin for histopathological evaluation. A second portion was used for the determination of oxidative stress biomarkers, while a third portion was reserved for the comet assay. The remaining tissue samples were snap-frozen in liquid nitrogen and stored at −80 °C for subsequent RNA extraction and molecular analyses.

#### 2.3.4. Assessment of Kidney Function

Renal function was evaluated by measuring serum urea and creatinine levels using commercially available diagnostic kits (Biodiagnostic, Giza, Egypt; Catalog numbers UR2110 and CR1251, respectively). All assays were performed according to the manufacturer’s instructions.

#### 2.3.5. Assessment of Oxidative Stress Markers

Kidney tissues were homogenized in phosphate buffer (pH 6.8) (El-Nasr Pharmaceutical Company, Cairo, Egypt) and centrifuged at 4000 rpm for 15 min at 4 °C. The supernatants were collected for the determination of malondialdehyde (MDA), glutathione peroxidase (GPx), and total antioxidant capacity (TAC) using commercially available diagnostic kits (Biodiagnostic, Giza, Egypt; Catalog Nos. MD2529, GP2524, and TA2513, respectively), according to the manufacturer’s instructions.

#### 2.3.6. RNA Extraction and Quantitative Real-Time PCR (qRT-PCR)

Total RNA was extracted from approximately 25–50 mg of frozen renal cortical tissue using the RNeasy Mini Kit (Qiagen, Hilden, Germany) according to the manufacturer’s instructions. RNA concentration and purity were determined spectrophotometrically using a NanoDrop 1000 spectrophotometer (Thermo Fisher Scientific, Wilmington, DE, USA), and samples with an A260/A280 ratio between 1.9 and 2.1 were used for subsequent analyses.

Gene expression was quantified by SYBR Green-based quantitative real-time PCR (qRT-PCR) using a StepOne™ Real-Time PCR System (Applied Biosystems, Foster City, CA, USA). Primer sequences are listed in Supplementary Table S1. Additional primer pairs were designed using Primer3 and FastPCR (v6.9.21) and validated for specificity and amplification efficiency prior to use.

Each 20 μL reaction mixture contained 10 μL of 2× HERA SYBR Green RT-qPCR Master Mix (Willowfort, Nottingham, UK), 1 μL of 20× RT Enzyme Mix, 1 μL each of forward and reverse primers (20 pmol), 3 μL of nuclease-free water, and 5 μL of RNA template. Amplification was performed with an initial denaturation step at 94 °C for 15 min, followed by 40 cycles of 95 °C for 15 s, 60 °C for 30 s, and 72 °C for 30 s, with a final extension at 72 °C for 10 min. Product specificity was confirmed by melt-curve analysis.

Relative gene expression was calculated using the 2^−ΔΔCt^ method as described by Livak and Schmittgen [20]. A housekeeping gene (β-actin) was used to normalize the expression data.

#### 2.3.7. DNA Methylation Analysis

Quantitative methylation-specific PCR (qMSP) was performed to assess Klotho promoter methylation as previously described [21,22]. Genomic DNA was extracted from renal tissue using the QIAamp DNA Mini Kit (Qiagen) according to the manufacturer’s instructions. DNA extraction and methylation analyses were conducted at the Biotechnology Unit, Animal Health Research Institute, Zagazig Branch, Egypt.

Bisulfite conversion of 500 ng genomic DNA was performed using the EZ DNA Methylation Kit (Zymo Research, Freiburg im Breisgau, Germany) according to the manufacturer’s protocol. Methylation-specific amplification was subsequently performed using the EpiTect MSP Kit (Qiagen, Hilden, Germany) with the bisulfite-converted DNA as the template.

Primer sets specific for methylated and unmethylated CpG sites within the rat Klotho promoter region were used for amplification. An input control primer set was included to normalize for variations in DNA input. The sequences of all primer pairs are listed in Supplementary Table S2 [23].

#### 2.3.8. Assessment of DNA Damage by Comet Assay

Renal tissue fragments were minced and suspended in chilled homogenization buffer (0.075 M NaCl and 0.024 M Na_2_EDTA, pH 7.5), followed by gentle homogenization on ice as previously described [24]. DNA strand break assessment was performed using an alkaline comet assay on standard microscope slides according to the method of Singh et al. [25]. Isolated nuclei were embedded in agarose on microscope slides and subjected to electrophoresis at 4 °C in the dark for 15 min at 25 V and approximately 250 mA. Slides were stained with 50 μL ethidium bromide (20 μg/mL) and examined using an Olympus BX51 fluorescence microscope (Olympus Corporation, Tokyo, Japan) at 400× magnification.

For each sample, 100 randomly selected nuclei were analyzed using CometScore™ software (version 1.5; TriTek Corp., Sumerduck, WV, USA). Cells exhibiting small heads and extensive fan-shaped tails were excluded, as these represent apoptotic nuclei rather than primary DNA damage. DNA damage was quantified by measuring tail length, percentage of DNA in the tail, and tail moment, with the latter two serving as the primary endpoints for comet assay analysis.

#### 2.3.9. Western Blotting and Protein Expression Analysis

Protein expression was evaluated using Western blotting as previously described [26]. Total protein was extracted from renal tissue using 1% SDS lysis buffer (El-Nasr Pharmaceutical Company, Cairo, Egypt) supplemented with protease and phosphatase inhibitor cocktails (Sigma-Aldrich, St. Louis, MO, USA). Histone proteins were isolated using an acid extraction protocol involving 0.4 N H_2_SO_4_ followed by neutralization with 1 N NaOH. Protein concentrations were determined using a bicinchoninic acid (BCA) assay kit (Thermo Fisher Scientific, Waltham, MA, USA).

Protein samples (50 μg total protein or 10 μg histone extract) were separated by 10% SDS-PAGE and transferred onto polyvinylidene difluoride (PVDF) membranes (Bio-Rad, Hercules, CA, USA). Membranes were blocked for 1 h at room temperature with 5% non-fat milk or 5% bovine serum albumin (BSA) in TBST and incubated overnight at 4 °C with primary antibodies against KIM-1 (ab47668, 1:1000), DNMT1 (#5032, 1:500), TNF-α (ab6671, 1:1000), H3K27me3 (#9733, 1:5000), H3K9me3 (#13968, 1:5000), and H3K4me3 (#9751, 1:5000).

After washing with TBST, membranes were incubated with horseradish peroxidase (HRP)-conjugated secondary antibodies for 1 h at room temperature. Immunoreactive bands were visualized using an enhanced chemiluminescence (ECL) detection system. Band intensities were quantified using ImageJ software (version 1.54p; National Institutes of Health, Bethesda, MD, USA) and normalized to β-actin or total histone H3 (#4499, 1:2000). Protein expression levels were expressed as fold change relative to the control group.

#### 2.3.10. Histopathological and Immunohistochemical Evaluation

Renal cortical tissues were fixed in 10% neutral-buffered formalin for 24 h, processed routinely, embedded in paraffin, sectioned at 3–5 μm, and stained with hematoxylin and eosin (H&E) for histopathological examination according to standard procedures [27].

For immunohistochemical detection of NF-κB, additional paraffin sections were mounted on positively charged slides and incubated with anti-NF-κB antibody (ab16502, 1:200 dilution; Abcam, Cambridge, UK) as previously described [28]. Both nuclear and cytoplasmic brown staining were considered positive.

Slides were examined and photographed using an Olympus microscope equipped with a digital camera at 400× magnification. Quantification of NF-κB immunoreactivity was performed using Image-Pro Plus software (version 6.0; Media Cybernetics Inc., Bethesda, MD, USA). For each sample, six randomly selected, non-overlapping fields were analyzed, and results were expressed as the percentage area of positive staining.

Semi-quantitative histopathological scoring was performed using six randomly selected fields per sample examined at 200× and 400× magnification, following a previously described modified method [29]. Histopathological changes were graded as absent (−), mild (+; 5–10% of examined tissue), moderate (++; 11–20%), or severe (+++; >20%). Evaluated parameters included tubular epithelial necrosis and dilatation, interstitial hemorrhage, edema, inflammatory cell infiltration, glomerular atrophy and congestion.

#### 2.3.11. Molecular Docking Analysis

RUT was docked against the STAT3 and TNF-α target proteins obtained from the Protein Data Bank (PDB IDs: 6NUQ and 2AZ5) to evaluate its binding affinity and molecular interactions [30,31].

The protein structures were prepared by removing co-crystallized ligands and water molecules, followed by the addition of hydrogen atoms and energy minimization [32]. RUT was similarly prepared by protonation, energy minimization, and conversion to the required PDBQT format [33]. Molecular docking was performed using AutoDock Vina version 1.5.7 [34]. The ligand was docked into the active site of each target protein, and the binding pose with the lowest binding energy was selected for further analysis. Binding affinity scores (kcal/mol) were recorded, and two-dimensional (2D) and three-dimensional (3D) protein–ligand interaction diagrams were generated using Discovery Studio Visualizer 2024 [35].

### 2.4. Statistical Analysis

Statistical analyses were performed using SPSS version 23 (IBM Corp., Armonk, NY, USA). Data are presented as mean ± SD. Differences among experimental groups were assessed using one-way analysis of variance (ANOVA), followed by Tukey’s post hoc test for pairwise multiple comparisons. Statistical significance was set at *p* < 0.05. Groups with different superscript letters indicate statistically significant differences. Since the investigated parameters represented distinct biological endpoints, including renal function, oxidative stress, inflammation, DNA damage, and epigenetic regulation, each endpoint was analyzed independently according to the predefined experimental comparisons, and no additional adjustment was applied across distinct biomarkers. Interpretation of the findings considered both statistical significance and the magnitude of biological changes to provide a balanced assessment of the observed effects.

## 3. Results

### 3.1. Characterization of RUT-NLCs Developed by Hot High-Shear Homogenization

**Figure 1:**
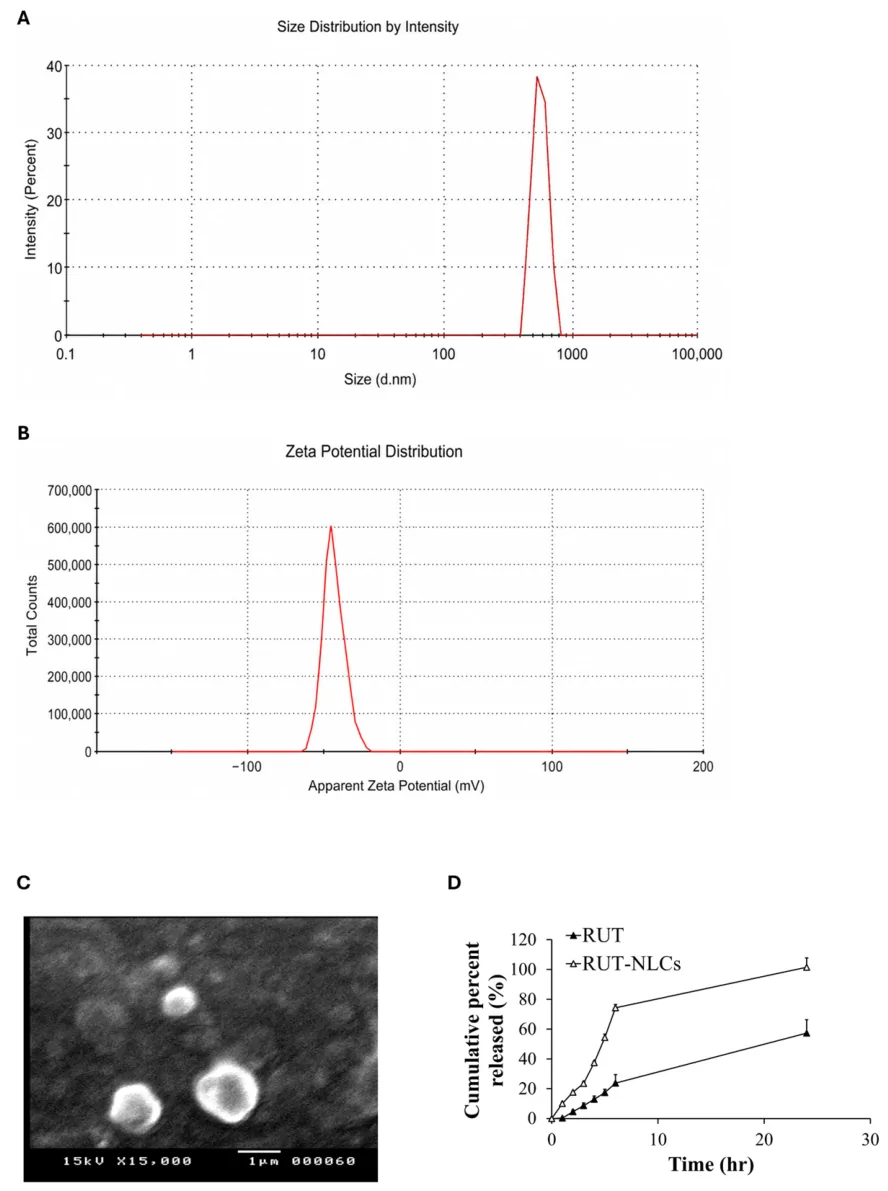
Characterization of the developed RUT-NLCs. RUT-loaded nanostructured lipid carriers (RUT-NLCs) were prepared using the hot high-shear homogenization method and characterized for (**A**) particle size, (**B**) zeta potential, (**C**) morphology by scanning electron microscopy (SEM), and (**D**) in vitro drug release profile.

Figure 1 presents the physicochemical characterization and in vitro release profile of the developed RUT-NLCs. Particle size analysis (Figure 1A) revealed a mean particle size of 566.9 ± 73.9 nm, indicating successful formation of nanoparticles within a size range favorable for enhanced stability, cellular uptake, and drug dissolution. The polydispersity index (PDI) of 0.294 indicated a relatively narrow particle size distribution, reflecting good homogeneity of the formulation. As shown in Figure 1B, the zeta potential was −43.7 ± 7.07 mV, indicating a highly negatively charged surface that is expected to promote colloidal stability by minimizing particle aggregation. Scanning electron microscopy (SEM) images (Figure 1C) demonstrated that the RUT-NLCs were predominantly spherical with smooth surfaces and a uniform morphology, supporting the successful preparation of the nanosystem. The in vitro release study (Figure 1D) showed significantly greater RUT release from the NLC formulation than from the pure drug. This enhanced release is likely attributable to the nanoscale particle size, increased surface area, and improved dispersion of RUT within the lipid matrix. Moreover, the release profile exhibited a biphasic pattern, characterized by an initial burst release followed by a sustained release phase, suggesting rapid release of surface-associated drug followed by gradual diffusion of the encapsulated drug from the lipid matrix. Collectively, these findings demonstrate that the developed RUT-NLCs possessed favorable physicochemical characteristics and an improved release profile, supporting their potential as an effective drug delivery system.

### 3.2. RUT-NLCs Attenuate DOX-Induced Renal Dysfunction

**Figure 2:**
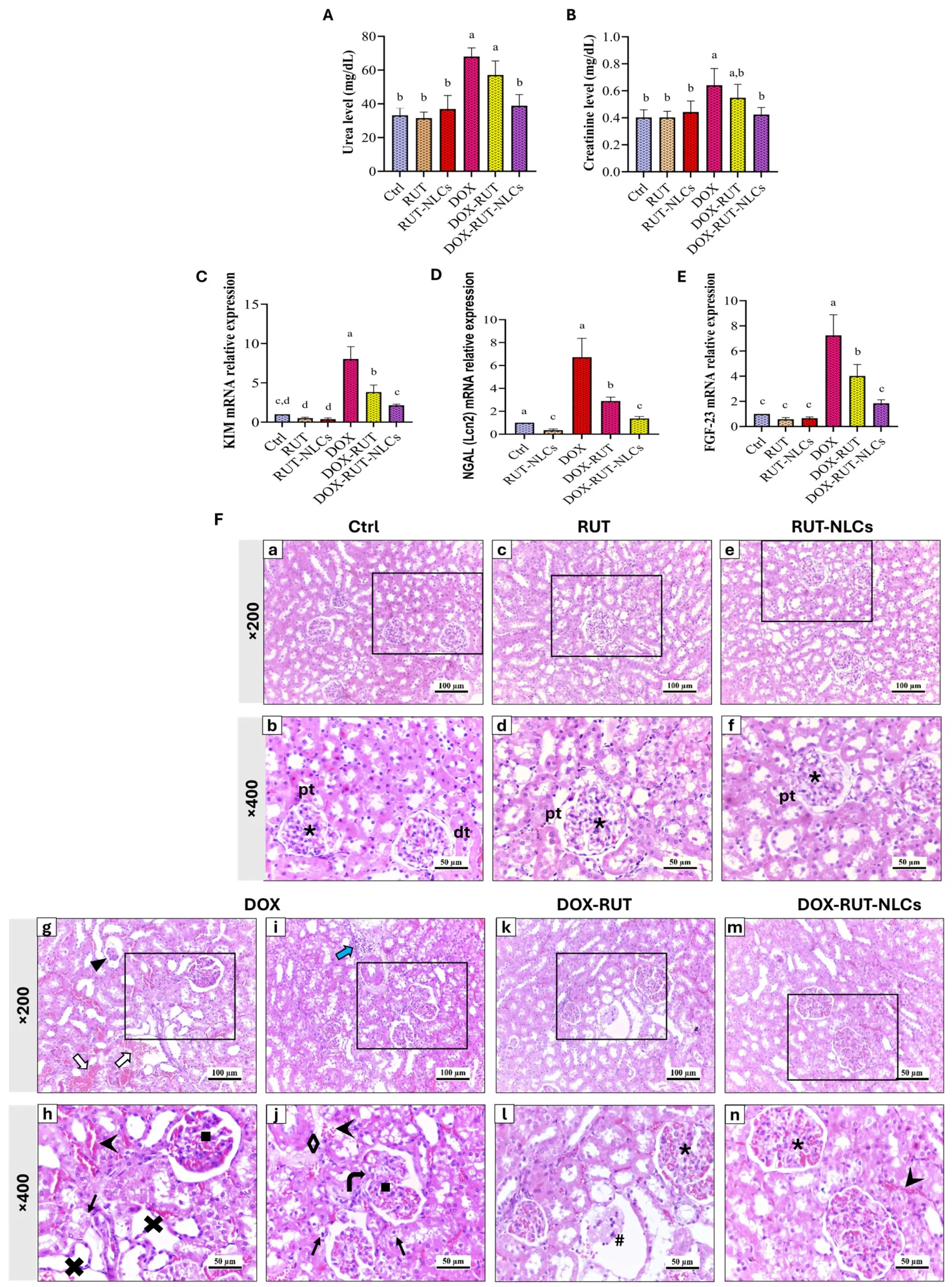
Effects of RUT-NLCs on Renal Function, Renal Injury Biomarkers, and Histopathological Alterations. Serum levels of (**A**) urea and (**B**) creatinine. Relative mRNA expression of (**C**) kidney injury molecule-1 (KIM-1), (**D**) neutrophil gelatinase-associated lipocalin (NGAL/Lcn2), and (**E**) fibroblast growth factor-23 (FGF-23). Data are presented as mean ± SD (*n* = 6). Different lowercase letters indicate statistically significant differences among groups (*p* < 0.05). (**F**) Representative hematoxylin and eosin (H&E)-stained sections of renal cortex from the experimental groups: (**a**,**b**) Ctrl; (**c**,**d**) RUT; (**e**,**f**) RUT-NLCs; (**g**–**j**) DOX; (**k**,**l**) DOX-RUT; and (**m**,**n**) DOX-RUT-NLCs. Panels (**a**,**c**,**e**,**g**,**i**,**k**,**m**) were acquired at ×200 magnification (scale bar = 100 µm). Boxed regions in panels (**a**,**c**,**e**,**g**,**i**,**k**,**m**) indicate areas shown at higher magnification in the corresponding panels (**b**,**d**,**f**,**h**,**j**,**l**,**n**) at ×400 magnification (scale bar = 50 µm). Representative histological features and histopathological alterations include normal proximal convoluted tubules (pt), distal convoluted tubules (dt), intact glomeruli (asterisk), congested glomeruli (square), hemorrhage (white arrows), inflammatory cell infiltration (blue arrow), dilated blood vessel with perivascular edema (◊), glomerular atrophy (▲), obliterated Bowman’s space (curved arrow), tubular epithelial degeneration with cytoplasmic vacuolation and pyknotic nuclei (thin arrows), extravasated erythrocytes (arrowheads), cystically dilated tubules (×), and interstitial widening with debris (#). Ctrl, control; RUT, rutin; RUT-NLCs, rutin-loaded nanostructured lipid carriers; DOX, doxorubicin; DOX-RUT, doxorubicin + rutin; DOX-RUT-NLCs, doxorubicin + rutin-loaded nanostructured lipid carriers.

The effects of RUT and RUT-NLCs on renal function were assessed by measuring serum urea and creatinine levels (Figure 2A,B). DOX administration significantly increased serum urea and creatinine levels by 105% and 67%, respectively, compared with the Ctrl group. Treatment with free RUT produced a modest, non-significant reduction in both biomarkers. In contrast, RUT-NLCs significantly decreased serum urea and creatinine levels by 43% and 35%, respectively, compared with the DOX group, with values approaching those of the Ctrl group. Moreover, RUT-NLCs produced a significantly greater reduction in serum urea than free RUT, while the difference in serum creatinine between the two treatments was not statistically significant. Neither RUT nor RUT-NLCs alone significantly affected serum urea or creatinine levels compared with the Ctrl group.

### 3.3. RUT-NLCs Attenuate DOX-Induced Upregulation of Acute Kidney Injury-Related Genes

The effects of RUT and RUT-NLCs on the mRNA expression of acute kidney injury biomarkers are presented in Figure 2C–E. DOX administration significantly upregulated the expression of KIM-1, NGAL (Lcn2), and FGF-23 by 7-, 5.7-, and 6.2-fold, respectively, compared with the Ctrl group. Free RUT treatment partially reduced the expression of these biomarkers; however, their levels remained significantly elevated relative to the Ctrl group. In contrast, RUT-NLCs produced a significantly greater reduction in the expression of all three genes, lowering their levels by approximately 75% compared with the DOX group and approaching those of the Ctrl group. Neither RUT nor RUT-NLCs alone significantly affected the expression of these biomarkers compared with the Ctrl group.

### 3.4. RUT-NLCs Attenuate DOX-Induced Histopathological Renal Injury

Examination of renal sections from the Ctrl group revealed normal cortical architecture, with intact renal corpuscles and tubular structures lined by healthy epithelial cells and no detectable histopathological alterations. The interstitium and renal vasculature also appeared unremarkable (Figure 2F, Ctrl). Similarly, kidneys from the RUT and RUT-NLC groups exhibited normal histological features comparable to those of the Ctrl group (Figure 2F, RUT and RUT-NLCs). In contrast, administration of a single dose of DOX (20 mg/kg) induced marked histopathological alterations characterized by extensive tubular necrosis with epithelial degeneration, vascular and glomerular congestion, glomerular atrophy, and prominent interstitial hemorrhage, consistent with acute tubular injury (Figure 2F, DOX). These histopathological changes were accompanied by the biochemical evidence of renal dysfunction. Treatment with free RUT partially improved the renal histological architecture, as evidenced by reduced tubular necrosis, decreased glomerular congestion, and less interstitial hemorrhage. However, focal areas of interstitial widening containing cellular debris remained evident (Figure 2F, DOX-RUT). In contrast, treatment with RUT-NLCs produced more pronounced histological improvement, with marked attenuation of the features of acute tubular injury. Only mild glomerular congestion and slight intertubular vascular congestion were observed (Figure 2F, DOX-RUT-NLCs).

Semi-quantitative histopathological scoring based on the lesions summarized in Table 1 further supported these observations. The Ctrl, RUT, and RUT-NLC groups showed no detectable histopathological lesions (score = 0). In contrast, the DOX group exhibited severe renal injury, with pathological changes affecting more than 21% of the examined tissue, including tubular necrosis and dilatation, glomerular congestion and atrophy, and interstitial edema with inflammatory cell infiltration. Treatment with free RUT reduced the severity of these lesions, resulting in predominantly mild-to-moderate histopathological scores compared with the DOX group. RUT-NLC treatment provided greater histological protection, with only mild pathological changes, including slight tubular necrosis and mild vascular congestion, indicating superior protection against DOX-induced renal injury.

### 3.5. RUT-NLCs Attenuate DOX-Induced Oxidative Stress

**Figure 3:**
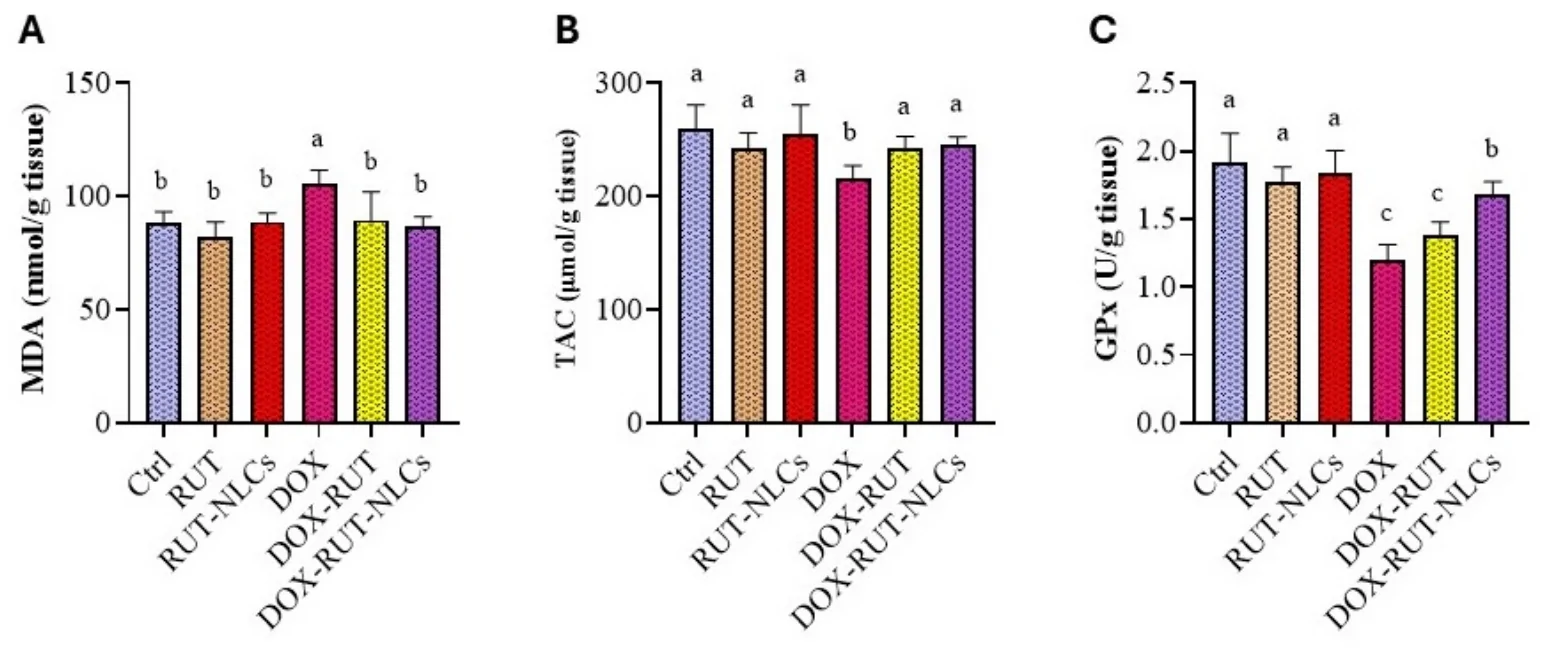
Effects of RUT-NLCs on Renal Oxidative Stress in DOX-Treated Rats. Renal levels of (**A**) malondialdehyde (MDA), (**B**) total antioxidant capacity (TAC), and (**C**) glutathione peroxidase (GPx) activity in the experimental groups. Data are presented as mean ± SD (*n* = 6). Different lowercase letters indicate statistically significant differences among groups (*p* < 0.05).

The effects of RUT and RUT-NLCs on renal oxidative stress are presented in Figure 3A–C. DOX administration significantly increased renal MDA levels by approximately 20% while reducing TAC and GPx activity by approximately 15% and 37%, respectively, compared with the Ctrl group. Treatment with free RUT reduced MDA levels by approximately 12% and restored TAC by approximately 11%, whereas the increase in GPx activity was not statistically significant. In contrast, RUT-NLCs produced a greater antioxidant effect, reducing MDA levels by approximately 18% and restoring TAC and GPx activity by approximately 14% and 40%, respectively, relative to the DOX group. Neither RUT nor RUT-NLCs alone significantly affected MDA, TAC, or GPx levels compared with the Ctrl group.

### 3.6. RUT-NLCs Attenuate DOX-Induced Renal Inflammation and MyD88/STAT3 Signaling

**Figure 4:**
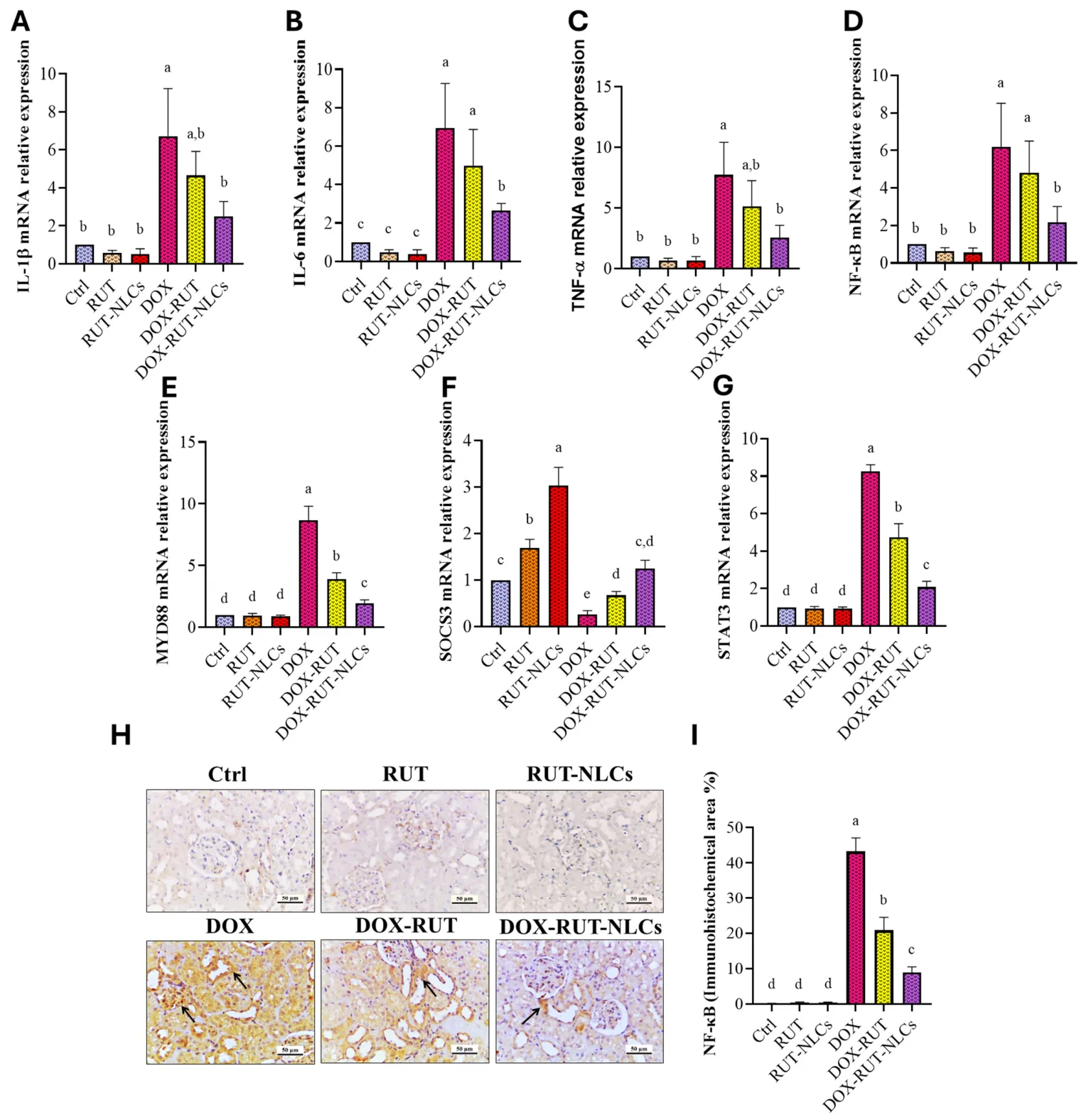
Effects of RUT-NLCs on Renal Inflammation and MyD88/STAT3 Signaling in DOX-Treated Rats. Relative mRNA expression of (**A**) interleukin-1β (IL-1β), (**B**) interleukin-6 (IL-6), (**C**) tumor necrosis factor-α (TNF-α), (**D**) nuclear factor-κB (NF-κB), (**E**) myeloid differentiation primary response 88 (MyD88), (**F**) suppressor of cytokine signaling 3 (SOCS3), and (**G**) signal transducer and activator of transcription 3 (STAT3) in the experimental groups. Data are presented as mean ± SD (*n* = 6). Different lowercase letters indicate statistically significant differences among groups (*p* < 0.05). (**H**) Representative immunohistochemical staining of NF-κB in renal cortical sections from the experimental groups (×400 magnification; scale bar = 50 μm). Positive immunoreactivity is indicated by brown nuclear and cytoplasmic staining (arrows). (**I**) Quantitative analysis of NF-κB immunoreactivity expressed as the percentage area of positive staining. Data are presented as mean ± SD (*n* = 6). Different lowercase letters indicate statistically significant differences among groups (*p* < 0.05). Ctrl, control; RUT, rutin; RUT-NLCs, rutin-loaded nanostructured lipid carriers; DOX, doxorubicin; DOX-RUT, doxorubicin + rutin; DOX-RUT-NLCs, doxorubicin + rutin-loaded nanostructured lipid carriers.

The effects of RUT and RUT-NLCs on renal inflammatory responses are presented in Figure 4A–D. DOX administration markedly increased the mRNA expression of IL-1β, IL-6, TNF-α, and NF-κB by approximately 6–7-fold compared with the Ctrl group, indicating pronounced inflammatory activation. Free RUT reduced the expression of these inflammatory markers by approximately 25–35% relative to the DOX group, whereas RUT-NLCs exerted a stronger inhibitory effect, decreasing their expression by approximately 60–65% compared with the DOX group and restoring levels close to those of the Ctrl group. Neither RUT nor RUT-NLCs alone significantly affected the expression of these markers compared with the Ctrl group. Collectively, RUT-NLCs demonstrated superior efficacy over free RUT in attenuating DOX-induced renal inflammatory gene activation.

The effects of different treatments on MyD88, SOCS3, and STAT3 mRNA expression are shown in Figure 4E–G. DOX administration markedly activated inflammatory signaling, as evidenced by an approximately 8-fold and 7-fold increase in MyD88 and STAT3 expression, respectively, along with a 75% reduction in SOCS3 expression compared with the Ctrl group. Free RUT partially reversed these alterations, decreasing MyD88 and STAT3 expression by approximately 55% and 40% and increasing SOCS3 expression by approximately 2.5-fold relative to the DOX group. Notably, RUT-NLCs exhibited a stronger regulatory effect, reducing MyD88 and STAT3 expression by approximately 75% and elevating SOCS3 expression by nearly 5-fold compared with the DOX group. Neither treatment significantly affected MyD88 or STAT3 expression in healthy rats, while both increased SOCS3 expression, indicating selective enhancement of anti-inflammatory regulation.

### 3.7. RUT-NLCs Attenuate Renal NF-κB Protein Expression in DOX-Treated Rats

Renal tissues from the control, RUT, and RUT-NLCs groups exhibited negligible NF-κB immunoreactivity, with no significant differences among them (*p* > 0.05) (Figure 4H, Ctrl, RUT, and RUT-NLCs). In contrast, DOX markedly increased NF-κB expression (*p* < 0.05), evidenced by intense nuclear and cytoplasmic immunostaining in tubular epithelial cells, predominantly within dilated tubules and, to a lesser extent, in the glomeruli (Figure 4H, DOX). RUT treatment significantly attenuated NF-κB expression relative to the DOX group (*p* < 0.05), resulting in moderate immunoreactivity largely confined to the dilated tubules (Figure 4H, DOX-RUT). In comparison, RUT-NLCs produced a more pronounced suppression of NF-κB expression, reducing immunoreactivity to mild levels that were significantly lower than those observed in both the DOX and DOX-RUT groups (*p* < 0.05) (Figure 4H, DOX-RUT-NLCs). Quantitative analysis of the immunopositive area is presented in Figure 4I.

### 3.8. RUT-NLCs Reverse DOX-Induced Alterations in DNA Methylation-Related Enzymes and Klotho Methylation

**Figure 5:**
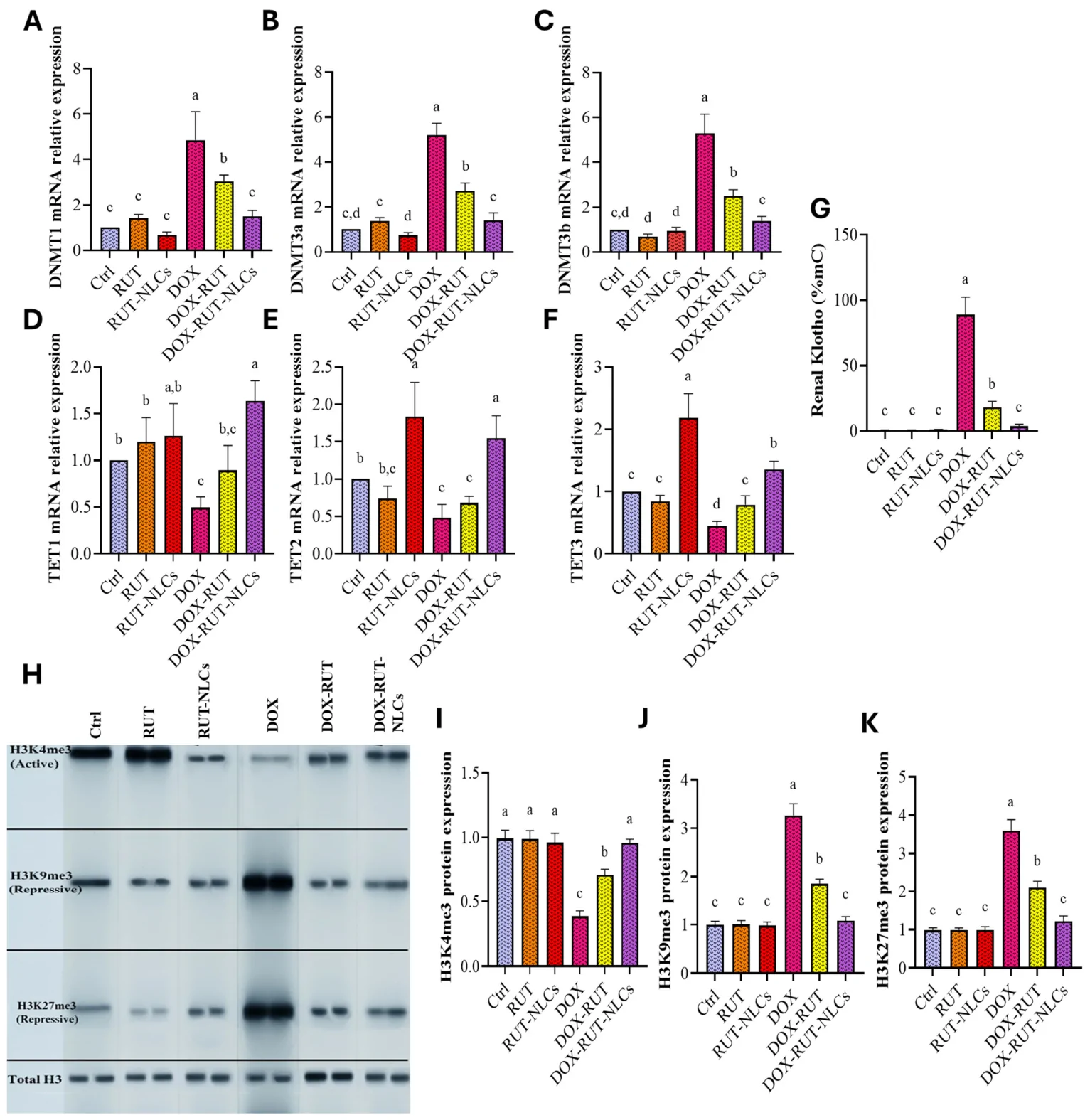
Effect of RUT-NLCs on renal DNA methylation and histone modifications in DOX-treated rats. Renal mRNA expression of (**A**) DNMT1, (**B**) DNMT3a, (**C**) DNMT3b, (**D**) TET1, (**E**) TET2, (**F**) TET3, and (**G**) Klotho in the experimental groups. (**H**) Representative Western blots showing the protein expression of H3K4me3, H3K9me3, H3K27me3, and total H3. Quantitative analysis of renal protein expression of (**I**) H3K4me3, (**J**) H3K9me3, and (**K**) H3K27me3. Data are presented as mean ± SD (*n* = 6). Bars with different letters are significantly different (*p* < 0.05).

Figure 5A–F illustrate the effects of DOX, RUT, and RUT-NLCs on renal DNA methylation-related enzymes. DOX administration markedly increased DNMT1, DNMT3a, and DNMT3b expression by approximately fourfold and reduced TET1, TET2, and TET3 expression by approximately 50% compared with the Ctrl group, indicating disrupted DNA methylation regulation. RUT partially restored these alterations, decreasing DNMT1, DNMT3a, and DNMT3b expression by approximately 40%, 50%, and 50%, respectively, while increasing TET1, TET2, and TET3 expression by approximately 80%, 40%, and 75%, respectively. RUT-NLCs exerted a stronger modulatory effect, reducing DNMT expression by approximately 70% and enhancing TET1, TET2, and TET3 expression by approximately twofold compared with the DOX group. Neither RUT nor RUT-NLCs alone significantly affected these epigenetic markers compared with the Ctrl group.

Figure 5G illustrates the effects of different treatments on renal Klotho methylation. DOX administration induced a marked increase in Klotho methylation, reaching approximately 123-fold above the Ctrl group. RUT co-treatment significantly reduced Klotho methylation by approximately 80% compared with the DOX group, although levels remained higher than controls. In contrast, RUT-NLCs produced a stronger demethylating effect, decreasing Klotho methylation by approximately 95% and restoring levels close to those of the Ctrl group. Neither RUT nor RUT-NLCs alone significantly altered Klotho methylation compared with the Ctrl group.

### 3.9. RUT-NLCs Restore Histone Methylation Patterns in the Renal Tissue of DOX-Treated Rats

Figure 5H presents representative Western blots of the histone methylation markers H3K4me3, H3K9me3, and H3K27me3. DOX administration disrupted histone methylation patterns, reducing H3K4me3 expression by approximately 60% and increasing H3K9me3 and H3K27me3 levels by approximately 2.3- and 2.7-fold, respectively, compared with the Ctrl group (Figure 5I–K). RUT partially restored these alterations, increasing H3K4me3 expression by approximately 85% and decreasing H3K9me3 and H3K27me3 levels by approximately 40% relative to the DOX group. Notably, RUT-NLCs exerted a stronger corrective effect, elevating H3K4me3 by approximately 150% and reducing H3K9me3 and H3K27me3 by approximately 65–70% compared with the DOX group. Neither RUT nor RUT-NLCs alone significantly altered these histone methylation markers compared with the Ctrl group.

### 3.10. RUT-NLCs Attenuate DOX-Induced TNF-α, DNMT1, and KIM-1 Protein Expression in Renal Tissue

**Figure 6:**
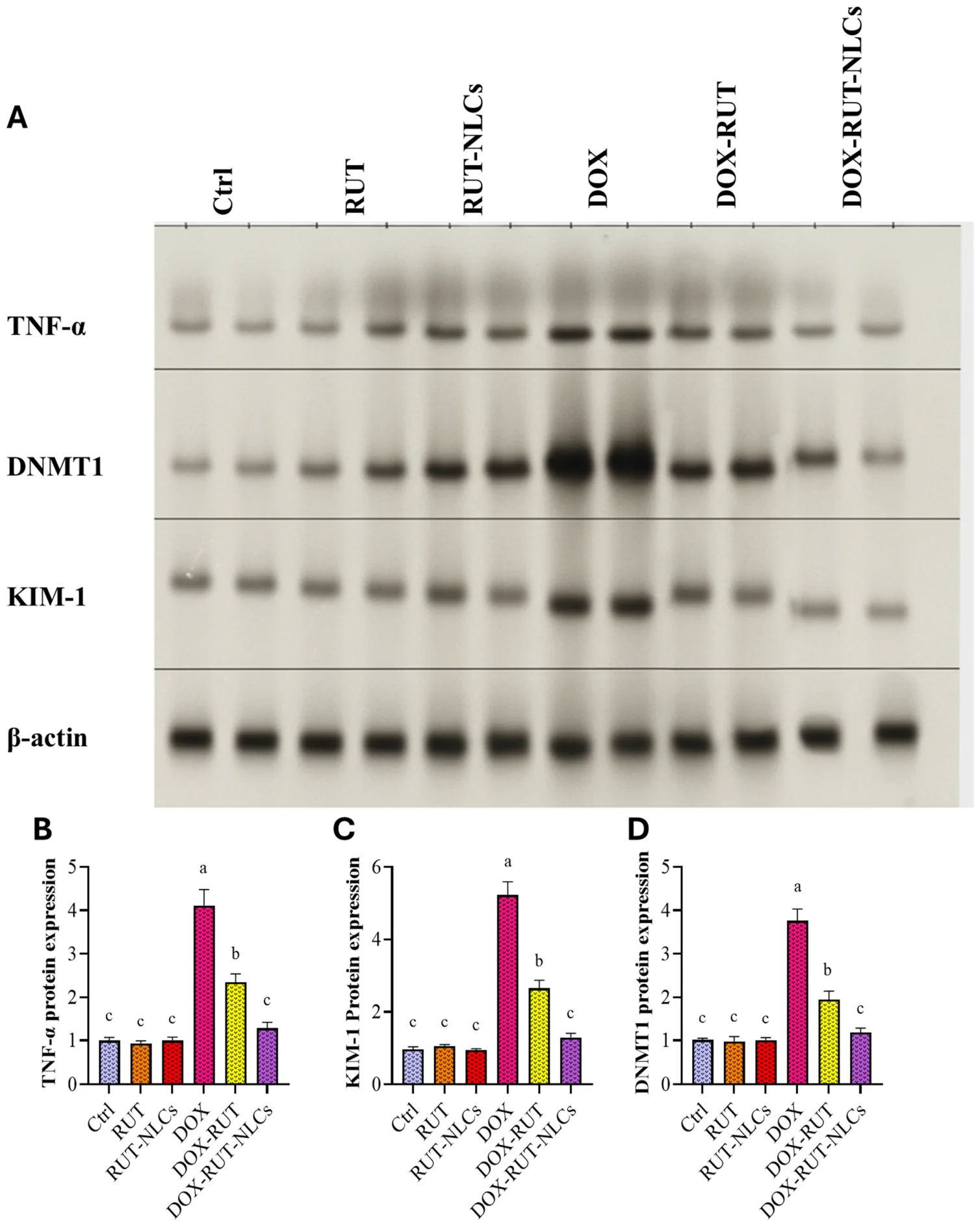
Effect of RUT-NLCs on renal TNF-α, DNMT1, and KIM-1 protein expression in DOX-treated rats. (**A**) Representative Western blots showing the protein expression of TNF-α, DNMT1, KIM-1, and β-actin in the experimental groups. Quantitative analysis of renal protein expression of (**B**) TNF-α, (**C**) DNMT1, and (**D**) KIM-1. Data are presented as mean ± SD (*n* = 6). Bars with different letters indicate statistically significant differences (*p* < 0.05).

DOX administration markedly increased TNF-α, DNMT1, and KIM-1 protein expression by approximately 4-, 3-, and 4.5-fold, respectively, compared with the Ctrl group (Figure 6A–D). RUT co-treatment partially attenuated these alterations, reducing the expression of the three proteins by approximately 40–50% relative to the DOX group. In comparison, RUT-NLCs produced a stronger protective effect, decreasing TNF-α, DNMT1, and KIM-1 expression by approximately 70% for all parameters, compared with the DOX group. Neither RUT nor RUT-NLCs alone significantly affected the expression of these proteins compared with the Ctrl group.

### 3.11. RUT-NLCs Attenuate DOX-Induced DNA Damage in Renal Tissue

**Figure 7:**
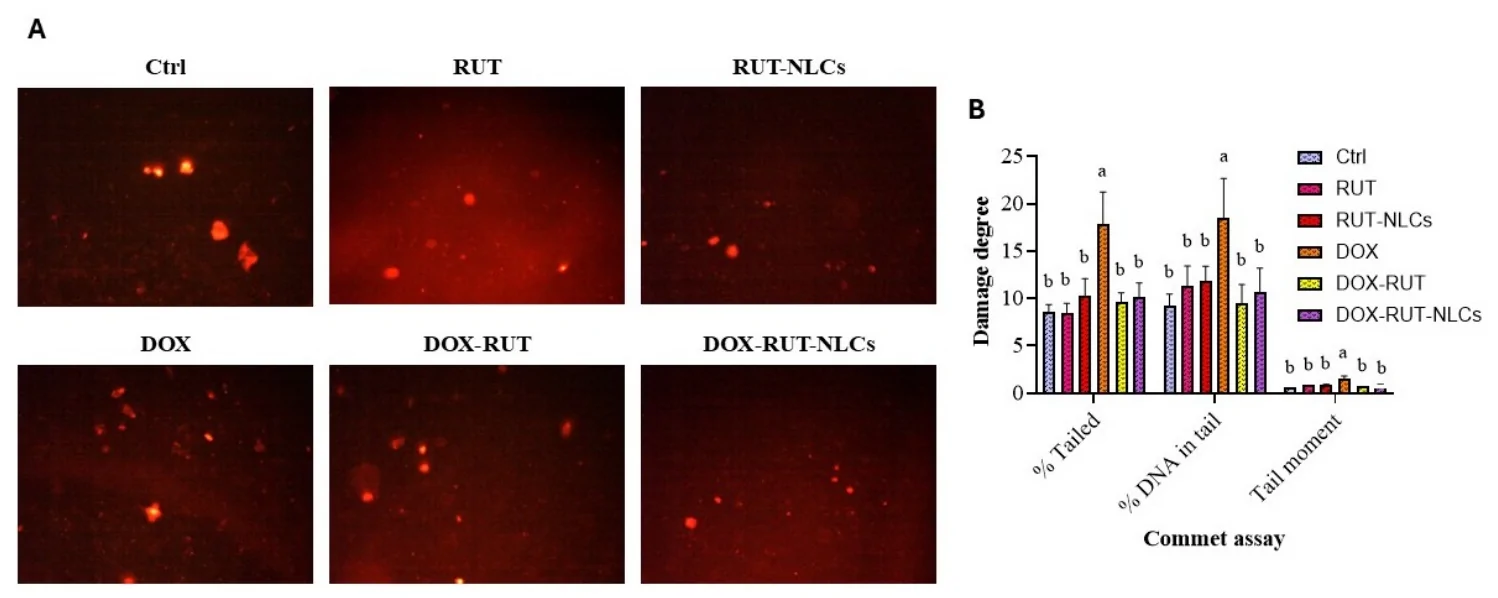
Effect of RUT-NLCs on renal DNA damage in DOX-treated rats. (**A**) Representative fluorescence micrographs of comet assay images from the experimental groups. (**B**) Quantitative analysis of comet assay parameters, including percentage of tailed DNA, percentage of DNA in the tail, and tail moment. Data are presented as mean ± SD (*n* = 6). Bars with different letters indicate statistically significant differences (*p* < 0.05).

DOX administration markedly increased DNA damage in renal tissue, as demonstrated by significant increases in the percentage of tailed DNA, tail DNA content, and tail moment compared with the control group (Figure 7). Co-treatment with RUT significantly attenuated these alterations, whereas RUT-NLCs produced a more pronounced reduction, restoring all comet assay parameters to levels comparable to those of the control group. Neither RUT nor RUT-NLCs alone significantly affected any comet assay parameter relative to the control group.

### 3.12. Molecular Docking Study

**Figure 8:**
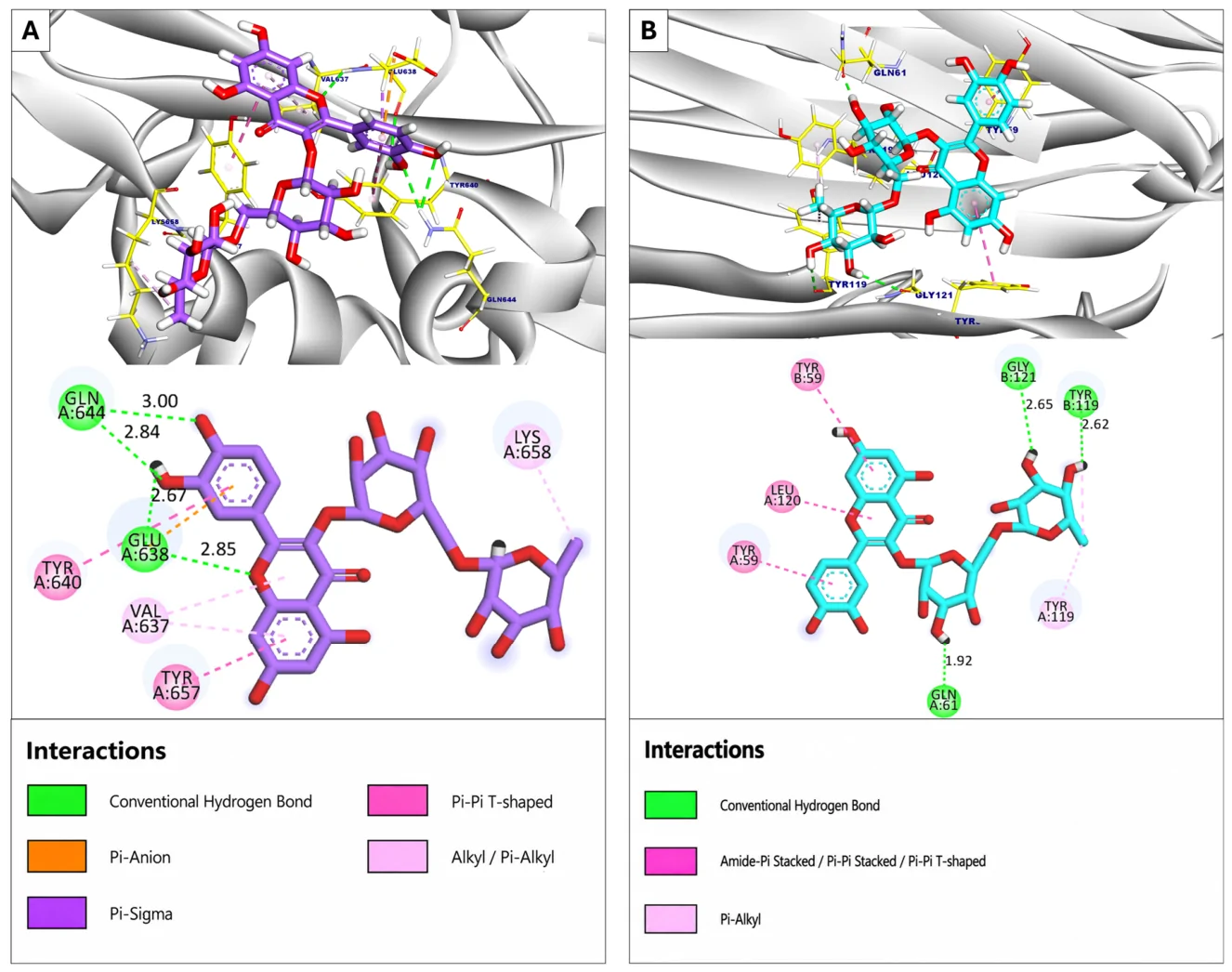
Molecular docking of RUT with STAT3 and TNF-α. (**A**) Three-dimensional representation of the predicted binding mode of RUT within the STAT3 binding pocket. Amino acid residues are shown in yellow, and RUT is shown in purple. (**B**) Three-dimensional representation of the predicted binding mode of RUT within the TNF-α binding pocket. Amino acid residues are shown in yellow, and RUT is shown in turquoise.

To investigate the potential molecular interactions underlying the observed biological effects, RUT was docked against the selected target proteins. The binding affinities of the highest-scoring docking poses are summarized in Table 2. RUT exhibited favorable binding toward STAT3, with a docking score of −8.03 kcal/mol. The interaction was stabilized by four hydrogen bonds with Glu638 (2.67 and 2.85 Å) and Gln644 (2.84 and 3.00 Å), together with six hydrophobic π-alkyl interactions involving Lys658, Tyr657, Val637, Tyr640, and Glu638 (Figure 8A). Likewise, RUT demonstrated favorable binding to TNF-α, with a docking score of −7.18 kcal/mol. The complex was stabilized by three hydrogen bonds with Gly121 (2.65 Å), Tyr119 (2.62 Å), and Gln61 (1.92 Å), in addition to five hydrophobic π-interactions involving Tyr119, Tyr59, and Leu120 (Figure 8B).

## 4. Discussion

The kidney is a highly specialized organ responsible for waste excretion, maintenance of systemic homeostasis, and regulation of acid–base balance [36]. Despite its broad clinical efficacy as an antineoplastic agent, doxorubicin (DOX) is frequently associated with dose-limiting nephrotoxicity, which restricts its therapeutic application [37]. Although several mechanisms have been implicated in DOX-induced renal injury, the precise molecular pathways underlying this toxicity remain incompletely understood [38]. In parallel, naturally occurring bioactive compounds have gained increasing attention because of their anti-inflammatory, anti-apoptotic, and antioxidant properties, making them promising candidates for the prevention of drug-induced organ injury [39]. Therefore, the present study investigated the nephroprotective effects of rutin (RUT) and RUT-loaded nanostructured lipid carriers (RUT-NLCs) against DOX-induced renal injury, with particular emphasis on the involvement of the MyD88/STAT3 signaling pathway and epigenetic regulation.

The prepared RUT-NLCs exhibited a relatively large particle size (~567 nm); nonetheless, they displayed favorable colloidal stability and retained effective biological activity. As particle size is a key determinant of nanocarrier biodistribution, cellular internalization, and pharmacokinetic behavior, further optimization toward smaller dimensions could enhance systemic performance and tissue-targeting efficiency. Nevertheless, larger lipid-based carriers offer certain advantages, including greater drug-encapsulation capacity and more sustained release kinetics, which may have contributed to the nephroprotective effects observed in the present study.

The present findings demonstrated that DOX administration resulted in marked renal dysfunction, as evidenced by significant elevations in serum urea and creatinine levels. These biochemical alterations were corroborated by histopathological evidence of renal injury, including tubular epithelial degeneration and necrosis, vascular and glomerular congestion, and extensive interstitial hemorrhage, all of which are characteristic features of DOX-induced acute tubular injury. Similar histopathological abnormalities have previously been attributed to oxidative stress and mitochondrial dysfunction, consistent with the observations of the present study [40]. In agreement with these findings, DOX also markedly disrupted renal redox homeostasis, as reflected by significant reductions in total antioxidant capacity (TAC) and glutathione peroxidase (GPx) activity, accompanied by a marked increase in malondialdehyde (MDA), a well-established marker of lipid peroxidation. Together, these findings indicate that DOX induces a profound oxidant–antioxidant imbalance in renal tissue and are consistent with previous observations reported by AlAsmari et al. [41].

Further evidence of DOX-induced acute kidney injury (AKI) was provided by the marked upregulation of the tubular injury biomarkers kidney injury molecule-1 (KIM-1) and neutrophil gelatinase-associated lipocalin (NGAL), both of which are well-established indicators of proximal tubular injury and early renal damage [42,43]. Likewise, fibroblast growth factor 23 (FGF23), a biomarker that is markedly elevated during AKI and has been implicated in phosphate homeostasis, was significantly increased following DOX administration [44]. Persistent elevation in FGF23 has also been associated with poor renal outcomes. Collectively, the increased expression of KIM-1, NGAL, and FGF23 further supports the ability of DOX to induce both functional and structural renal injury, consistent with previous findings reported by Gad et al. [45]. In contrast, pretreatment with either RUT or RUT-NLCs markedly attenuated these alterations, with RUT-NLCs providing greater protection. This improvement was accompanied by restoration of renal function, recovery of antioxidant defenses, as evidenced by increased TAC and GPx together with reduced MDA levels, and significant reductions in KIM-1, NGAL, and FGF23, indicating effective protection against both tubular and glomerular injury.

Within mitochondria, DOX undergoes redox cycling to generate an unstable semiquinone intermediate, which reacts with molecular oxygen to produce excessive reactive oxygen species, thereby promoting oxidative injury in renal tissue [46,47]. The kidney is particularly susceptible to oxidative damage because of its high metabolic activity, abundant mitochondrial content, and lipid-rich cellular membranes, all of which favor reactive oxygen species generation and oxidative DNA damage [48]. Consistent with this mechanism, the comet assay demonstrated marked DNA damage in DOX-treated kidneys, as evidenced by increased tailed DNA, tail DNA content, and tail moment. In addition to oxidative stress, DOX has been shown to directly intercalate into DNA and inhibit topoisomerase II, thereby impairing DNA repair and promoting double-strand DNA breaks [49]. Pretreatment with RUT-NLCs significantly attenuated all comet assay parameters, indicating substantial protection against DOX-induced genotoxicity. These findings are consistent with previous reports demonstrating that nanoformulations of natural antioxidants mitigate DOX-induced DNA damage in renal tissue [50]. The enhanced protection afforded by RUT-NLCs may reflect improved drug delivery; however, enhanced bioavailability remains a proposed explanation that requires pharmacokinetic confirmation [51].

DNA methylation is a fundamental epigenetic mechanism that regulates gene expression during mammalian development [52] and has been implicated in the pathogenesis of several renal disorders, including chronic kidney disease and diabetic nephropathy [53]. In the present study, DOX administration markedly disrupted the DNA methylation machinery, as evidenced by the upregulation of DNMT1, DNMT3a, and DNMT3b, together with the downregulation of TET1, TET2, and TET3. These alterations were substantially attenuated by both RUT and RUT-NLCs, with the nanoformulation demonstrating greater efficacy in restoring the expression of these epigenetic regulators. These findings suggest that RUT, particularly when delivered as an NLC formulation, effectively mitigates DOX-induced epigenetic dysregulation, potentially in association with its antioxidant properties [54]. Our findings are consistent with previous reports demonstrating that oxidative stress promotes aberrant DNA methylation through activation of DNA methyltransferases. Zhao et al. reported that excessive ROS enhance DNA methyltransferase activity, resulting in abnormal DNA hypermethylation that contributes to renal fibrosis [55]. Similarly, oxidative stress has been recognized as a major driver of epigenetic remodeling, including alterations in DNA methylation and chromatin organization, in a variety of pathological conditions such as cancer and cardiac fibrosis [54,56,57]. Mechanistically, elevated ROS activate hypoxia-inducible factor-1α (HIF-1α), which increases the expression of DNA methyltransferases and promotes global DNA hypermethylation. At the same time, ROS suppress the activity of TET enzymes, thereby impairing DNA demethylation and further disrupting gene expression [58].

Klotho promoter hypermethylation has been implicated in reduced Klotho expression in kidney injury and may contribute to the loss of its renoprotective effects [10]. Consistent with these observations, the present study demonstrated marked hypermethylation of the Klotho promoter following DOX administration, which was substantially reversed by RUT and, more effectively, by RUT-NLCs. Previous studies have similarly shown that inhibiting DNMT1- and DNMT3a-mediated methylation can restore endogenous Klotho expression and improve renal protection, highlighting the therapeutic potential of epigenetic modulation in kidney disease [59]. Previous studies have also reported associations between reduced Klotho expression and enhanced inflammatory signaling. Klotho protected against Van-induced AKI by inactivating the JAK2/STAT3 signaling pathway. Moreover, exogenous Klotho has been reported to increase SOCS3 expression, a well-recognized negative regulator of STAT3 activation [60,61,62]. In parallel, reduced Klotho expression enhances MyD88-dependent signaling, thereby amplifying Toll-like receptor-mediated inflammatory responses and promoting renal fibrosis and apoptosis [63]. Together, these findings from different experimental models provide a rationale for examining Klotho and MyD88/STAT3 signaling concurrently. However, they do not establish that Klotho epigenetic regulation directly controls MyD88/STAT3 signaling in DOX-induced renal injury.

Consistent with the observed epigenetic alterations, DOX administration induced marked hypermethylation of the Klotho promoter, associated with activation of the MYD88/STAT3 signaling pathway and reduced SOCS3 expression. Based on the present findings, we propose that epigenetic repression of Klotho may represent one mechanism associated with dysregulation of the MYD88/SOCS3/STAT3 axis during DOX-induced AKI. However, further functional studies are required to establish a direct mechanistic relationship. Notably, treatment with either RUT or RUT-NLCs largely reversed these alterations by restoring Klotho expression, increasing SOCS3 expression, and suppressing MYD88/STAT3 activation, with the nanoformulation exhibiting greater efficacy. These findings highlight the ability of RUT, particularly in its NLC formulation, to attenuate DOX-induced renal injury alongside improvements in epigenetic and inflammatory signaling markers.

In addition to DNA methylation, histone methylation represents another major epigenetic mechanism regulating the renal response to injury. Among the best-characterized histone modifications, H3K4me3 is generally associated with transcriptional activation, whereas H3K9me3 and H3K27me3 are linked to transcriptional repression. In the present study, DOX profoundly disrupted renal histone methylation patterns, as evidenced by marked increases in H3K9me3 and H3K27me3 together with a reduction in H3K4me3. Such alterations are associated with a more transcriptionally repressive chromatin state and may contribute to reduced expression of genes involved in antioxidant defense, mitochondrial function, and cellular repair [5,64]. The observed increases in H3K9me3 and H3K27me3 may reflect enhanced histone methyltransferase activity under conditions of oxidative stress and inflammation [65], whereas the reduction in H3K4me3 may contribute to the suppression of renoprotective and anti-apoptotic genes, thereby exacerbating oxidative stress and tubular cell injury [66].

These findings are consistent with previous reports demonstrating that dysregulated histone methylation contributes to the progression of cisplatin-induced AKI [67]. Importantly, both RUT and RUT-NLCs effectively restored the balance of renal histone methylation, with the nanoformulation producing a more pronounced effect. Specifically, treatment increased H3K4me3 while reducing H3K9me3 and H3K27me3, thereby promoting a more transcriptionally permissive chromatin environment. Restoration of this epigenetic landscape may facilitate the expression of genes involved in cellular repair, maintenance of mitochondrial integrity, and antioxidant defense [68,69]. In addition, enrichment of H3K4me3 may enhance the transcription of Nrf2-regulated antioxidant genes, including HO-1, thereby limiting ROS accumulation and apoptosis [70]. Simultaneously, suppression of H3K9me3 and H3K27me3 may attenuate pro-inflammatory and profibrotic pathways, including NF-κB signaling, ultimately preserving renal structure and function [71]. Collectively, these findings indicate that RUT modulates DOX-induced alterations in histone methylation, providing an additional potential mechanism associated with its renoprotective effects.

The superior renoprotective effects of RUT-NLCs compared with free RUT may reflect several advantages of the nanostructured lipid carrier system, including improved aqueous dispersibility, protection of rutin from degradation, and sustained drug release. These properties could increase rutin exposure in renal tissue and contribute to the observed biological effects. However, because pharmacokinetic, biodistribution, and tissue concentration studies were not performed, enhanced bioavailability cannot be established as the basis for the greater efficacy of RUT-NLCs. Future studies should therefore characterize plasma pharmacokinetics, tissue distribution, and renal rutin concentrations to determine how formulation properties influence drug exposure and nephroprotective activity.

From a translational perspective, the present findings support further investigation of RUT-NLCs as a potential adjunctive approach to limit renal injury during DOX-based chemotherapy, although several aspects require further evaluation to establish their clinical feasibility. Manufacturing procedures should be optimized and standardized to ensure scalability, batch-to-batch reproducibility, and consistent physicochemical properties. Long-term stability studies under different storage conditions are also needed to establish formulation integrity, appropriate storage requirements, and shelf life. In addition, comprehensive pharmacokinetic, biodistribution, safety, excipient compatibility, and immunogenicity studies will be important for further development. Studies in tumor-bearing models are particularly important to determine whether RUT-NLCs can preserve renal function without compromising the antitumor efficacy of DOX.

Several limitations of the present study also identify important directions for future investigation. Although changes in epigenetic markers, Klotho expression, and MyD88/STAT3 signaling were observed concurrently, these findings establish an association rather than a direct causal relationship among these events. Functional studies using targeted epigenetic manipulation, Klotho silencing or overexpression, and selective inhibition or genetic modulation of MyD88 and STAT3 are needed to clarify their mechanistic relationship. Similarly, molecular docking provides theoretical evidence of potential interactions between rutin and selected molecular targets but does not confirm functional interactions or biological activity in vivo. These predicted interactions should therefore be validated using appropriate biochemical and molecular approaches. In addition, the long-term physicochemical stability of the RUT-NLC formulation was not evaluated. Systematic stability studies assessing particle size, polydispersity index, zeta potential, drug content, and release characteristics during storage would help establish formulation robustness and reproducibility.

The relatively small sample size and exclusive use of young male animals may limit the generalizability of the findings. Because sex- and hormone-related differences may influence DOX-induced renal injury as well as inflammatory and epigenetic responses, future studies should include both sexes and animals of different ages to determine whether the observed effects are consistent across these biological variables. In addition, the study employed a single acute model induced by one dose of DOX. Although this model is suitable for examining early renal injury, it does not address the persistence of RUT-NLC-mediated protection during renal recovery or under cumulative DOX exposure. Future studies should therefore include chronic and cumulative-dose models that more closely reflect clinical patterns of DOX administration, together with longitudinal assessment of renal function and associated molecular and epigenetic changes. Finally, larger cohorts, dose–response studies, pharmacokinetic and biodistribution analyses, and subsequent clinical investigations will be necessary to further establish the therapeutic and translational potential of RUT-NLCs.

## 5. Conclusions

The present study demonstrates that doxorubicin-induced acute kidney injury is accompanied by oxidative stress, epigenetic dysregulation, and activation of the MyD88/STAT3 signaling pathway. RUT-NLCs provided superior renoprotection compared with free RUT, characterized by preservation of renal function and histological architecture, restoration of redox homeostasis, attenuation of DNA damage, improvement in aberrant DNA and histone methylation, and suppression of MyD88/STAT3-related inflammatory signaling. These coordinated molecular changes suggest a potential mechanistic interplay among epigenetic remodeling, Klotho regulation, and inflammatory signaling; however, the causal relationships among these processes require further investigation.

## Figures and Tables

**Table 1:** Semi-Quantitative Histopathological Scoring of Renal Cortical Lesions.

		Ctrl	RUT	RUT-NLCs	DOX	DOX-RUT	DOX-RUT-NLCs
Tubules	Necrosis	−	−	−	+++	++	+
	Dilatation	−	−	−	+++	++	+
Interstitium	Hemorrhage	−	−	−	+++	+	+
	Edema	−	−	−	++	+	−
	Inf. Cells	−	−	−	++	+	−
Glomeruli	Atrophy	−	−	−	++	+	−
	Congestion	−	−	−	+++	++	+

Histopathological lesions were graded as absent (−), mild (+; 5–10% of the examined tissue), moderate (++; 11–20% of the examined tissue), or severe (+++; >20% of the examined tissue).

**Table 2:** Molecular Docking Analysis of RUT against STAT3 and TNFα.

Target Proteins	RUT	
	RMSD Value (Å)	Affinity Score (kcal/mol)
STAT3	1.21	−8.03
TNFα	1.54	−7.18

## Data Availability

The data supporting the findings of this study are available from the authors upon reasonable request.

## Supplementary Materials

The following supporting information can be downloaded at https://www.mdpi.com/article/10.3390/toxics14080713/s1, Table S1: Primers, amplification conditions, and amplicon sizes for gene expression analysis [72,73]; Table S2: Primer sequences for methylation-specific PCR. Table S3: Primary antibodies used for Western blotting.
