# Supplementary material for: Rutin-Loaded Nanostructured Lipid Carriers Attenuate Doxorubicin-Induced Nephrotoxicity and Modulate Epigenetic Regulation and MyD88/STAT3 Signaling
Source: Toxics. 2026 Aug 12;14(8):713. doi: 10.3390/toxics14080713 (PMC13517413; doi:10.3390/toxics14080713)
Supplement: Supplementary file 1 [file toxics-14-00713-s001.zip › toxics-4441245-supplementary.pdf]

Table S1: Primers, Amplification Conditions, and Amplicon Sizes for Gene Expression Analysis

Target gene	Primers sequences (5′ → 3′)	GenBank accession no.
**STAT3**	F: CTGCCCCTTACCTGAAGACC	NM_001430048.1
	R: TCCATGTCAAACGTGAGCGA	
**MYD88**	F: CGGAGGAGATGGGTTTCGAG	NM_198130.2
	R: CTCGATGCGGTCCTTCAGTT	
**SOCS-3**	F: CCTTTTCCAACGCCAAAGGG	NM_053565.2
	R: ATCCCCATCCATGGGACAGA	
	**DNA methylation**	
**DNMT1**	F: GCCCCATGAAACGCTCTAAG	NM_053354.3
	R: TTGCCACCTTTCTTCTTGCC	
**DNMT3a**	F: AGAAGCCGCTGTTACCTCTT	XM_032908615.1
	R: GCTGAAACCCTTTGCACAGA	
**DNMT3b**	F: TGAGAAGCCAGGACCAGAAG	NM_001396349.1
	R: TCATTTCCAGTGATGCCCCT	
**TET1**	F ACATTGCTGGAGACTGTCGA	XM_032888331.1
	R: TTCTGTCACGGCCATCTTCT	
**TET2**	F: CAGAACGGAAGCCCATTAGC	NM_001427557.1
	R: CTGTGCGTTTTATCCCTCCG	
**TET3**	F: GCAAGGAGGGGAAGAGTTCT	XM_032906253.1
	R: GGAGGGTATCAGTGAGCTCC	

**Kidney injury**		
**KIM-1**	F: CACTCACTGCAGCGATTCTC	AF035963.1
	R: ATGCTCCCATGATTTTGCCC	
**NGAL (Lcn2)**	F: AACGTCACTTCCATCCTCGT	NM_130741.2
	R: CCATGGCAAACTGGTCGTAG	
**FGF-23**	F: AAACTCAAGCCCAACACCAC	NM_130754.2
	R: CTCAAGGACAGAAGTGGGGT	
**Inflammation**		
**IL-1β**	F: CACCTCTCAAGCAGAGCACAG	[72]
	R: GGGTTCCATGGTGAAGTCAAC	
**IL-6**	F: TCCTACCCCAACTTCCAATGCTC	[72]
	R: TTGGATGGTCTTGGTCCTTAGCC	
**TNF-α**	F: AAATGGGCTCCCTCTCATCAGTTC	[72]
	R: TCTGCTTGGTGGTTTGCTACGAC	
**NF-κB**	F: AATTGCCCCGGCAT	[73]
	R: TCCCGTAACCGCGTA	
**House keeping**		
**β- actin**	F: CAACCTTCTTGCAGCTCCTC	KJ696744.1
	R: ATACCCACCATCACACCCTG	

Table S2. Primer sequences for methylation-specific PCR.

Primer Name	Sequence	Purpose
**mKL/M**	(5′→3′): GGTATCGCGGGTATTTTTAATC	Detects methylated Klotho DNA
	(3′→5′): CGACATAATCCCTAAAATAATCGAC	
**mKL/unM**	(5′→3′): TTAATGGTATTGTGGGTATTTTTAATTG	Detects unmethylated Klotho DNA
	(3′→5′): CAACATAATCCCTAAAATAATCAAC	
**mIn**	(5′→3′): TAGTTTTAGGAAGGTAAAGGGAGTG	Normalization Control
	(3′→5′): AAATCCCAAAAAAAACACAACAAA	

Table S3: Primary antibodies used for western blotting:

Target Protein	Primary Antibody Source	Common Catalog No.	Dilution	Loading Control
**KIM-1**	Rabbit anti-KIM-1	Abcam (ab47668)	1:1000	β-actin
**DNMT1**	Rabbit anti-DNMT1	CST (#5032)	1:500	β-actin
**TNF-α**	Rabbit anti-TNF-α	Abcam (ab6671)	1:1000	β-actin
**H3K27me3**	Rabbit anti-H3K27me3	CST (#9733)	1:5000	Total Histone H3
**H3K9me3**	Rabbit anti-H3K9me3	CST (#13968)	1:5000	Total Histone H3
**H3K4me3**	Rabbit anti-H3K4me3	CST (#9751)	1:5000	Total Histone H3
**β-actin**	Mouse anti-β-actin	Santa Cruz (sc-47778)	1:10,000	N/A
**Total Histone H3**	Rabbit anti-Total H3	CST (#4499)	1:2000	N/A
